# Validation of the Greek translation of the multicultural quality of life index (MQLI-gr)

**DOI:** 10.1186/s12955-020-01426-9

**Published:** 2020-06-15

**Authors:** Effrosyni D. Kokaliari, Ann W. Roy

**Affiliations:** Department of Graduate Social Work Springfield, Springfield College School of Social Work and Behavioral Sciences, 263 Alden Street, Springfield, MA 01109 USA

**Keywords:** Construct validity, Greek translation, Confirmatory factor analysis, Measurement

## Abstract

**Background:**

The aim of the current study was to examine the internal structure and convergent and discriminant validity of the *Multicultural Quality of Life Index* (MQLI) in a Greek sample of community-dwelling adults in a major Greek city.

**Methods:**

The authors developed a Greek version of the *Multicultural Quality of Life Index* (*MQLI-Gr*). It was translated following cross-cultural adaptation procedures for self-report measures and administered to community members (*N* = 884). Participants completed a *brief demographic survey*, the *MQLI-Gr*, and the *Depression Anxiety Stress Scales* (DASS-42).

**Results:**

The *MQLI-Gr* is brief, easy to use, and demonstrates strong internal consistency (Cronbach alpha = .90). In terms of internal structure there were mixed results. In terms of discriminant validity, statistically significant differences in mean *MQLI-Gr* scores were observed between two groups: those with *none-mild* symptoms versus those with *severe symptoms* of depression, anxiety, and stress (*p* < .05). The *MQLI-Gr* was also able to discriminate among groups assumed to vary on quality of life; marital status, income, and employment. In terms of convergent validity, results were in the expected direction, with participants reporting high levels of depression, anxiety, and stress, also reporting lower quality of life on the *MQLI-Gr* (*p* < .001).

**Conclusion:**

Consistent with other translations, the *MQLI-Gr* demonstrated feasibility, strong internal consistency, and good convergent and discriminant validity. This is the first step in the development of a psychometrically sound measure to assess *quality of life* in a community-dwelling population in Greece. With the addition of further validation studies, this measure will be a useful tool for assessing the *quality of life* in the Greek community.

## Background

The aim of the current study was to validate a Greek translation of the *Multicultural Quality of Life Index* (MQLI) using a sample of community-dwelling adults in a major city in Greece. Following the 2008 economic crisis and for several years thereafter there was a precipitous decline in the Greek quality of life. An increase in rates of unemployment, poverty, depression, and suicide [1–6], as well as severe cuts to workers’ salary, pensions, and health care [7, 8] are well-documented. Such factors contributed to a significant drop in the Greek standard of living. Although the primary aim of the current study was to validate a Greek translation of the MQLI, we were initially motivated by the impact of the economic crisis in Greece on mental health and quality of life [9].

To assess construct validity, and in particular, convergent and discriminant validity, three hypotheses were posed as follows: H_1_-the higher the score on depression, anxiety, and stress the lower the score on *MQLI-Gr*; H_2_-*MQLI-Gr* will distinguish between participants with ‘none-mild symptoms’ of depression, anxiety, and stress versus those with ‘severe symptoms’; and H_3_-*MQLI-Gr* will distinguish among groups assumed to vary on quality of life: marital status, income, and employment. Specifically, single status, low income, and being unemployed will be inversely related to quality of life. To examine internal structure, a fourth hypothesis was posed: H_4_-using a one-factor structure, the latent variable *Personal Satisfaction* will be explained by 10 indicator variables of the *MGLI-Gr* in a confirmatory factor analysis (CFA). It is the hope of the authors that the current validation study of the *MQLI-Gr*, along with future such studies, will enable demographers and social service providers to accurately document and track changes in the quality of life in Greek communities.

### Limitations of quality of life scales

The concept *quality of life* has been widely applied in the field of medicine. Over the years, the phrase *quality of life* has broadened to include social functioning and well-being and it is now common to find *quality of life* measures in social and epidemiological studies [9, 10]. Numerous quality of life measures have been translated and validated in the Greek language including: Quality of Life in Epilepsy (QOLIE-31) [11], the Minnesota Life with Heart Failure Questionnaire (MLWHFQ) [12], the Stroke and Aphasia Quality of Life Scale (SAQOL-39 g) [13], the Transfusion Dependent for Thalassemia Quality of Life Questionnaire (TRANQoL) [14], and the Macular Degeneration Quality of Life (MacDQoL) [15]. The aforementioned QoL Greek translations have been used to examine the impact of heart disease, stroke, and other chronic health conditions on *quality of life* [11–15]; however, they do not address overall health and social functioning [10], nor are they appropriate for use with the general population.

The M*ulticultural Quality of Life Index (*MQLI) is a broad based scale and was originally developed in response to a dearth of culturally relevant measures to assess *quality of life* [10, 16-18]. The MQLI is a 10-item, self-report measure with items rated on a scale from one to ten (poor to excellent). It takes about 3 min to complete and is used to assess ten dimensions of life satisfaction: physical well-being; psychological/emotional well-being; self-care and independent functioning; occupational functioning; interpersonal functioning; social emotional support; community and services support; personal fulfillment; spiritual fulfillment; and overall quality of life [10]. The ten items were developed based on thematic analysis of identified dimensions in relevant international literature. In the literature it is described as one of the most comprehensive measures in its scope as it includes inquiries that range from physical well-being to spirituality. The MQLI demonstrates strong test-retest reliability (*r* = .87) among two cross-ethnic English samples [10] and has been validated in several languages including: Korean, Chinese and Spanish [16, 17, 193, 20]. Translated versions report strong internal consistency and test-retest reliability [e.g., English, α = 0.92, *r* = .87 [10]; Korean, α = 0.97*, r* = .85 [16]; Chinese, α = 0.94, *r* = .80 [17]; Spanish, α = 0.88, *r* = .94 [20]. The added value of the current study is that the *MQLI-Gr*, unlike prior QoL Greek translations, includes a broad set of health and social indicators. In addition, to the best of our knowledge, there are no Greek translations of the MQLI that have been validated on a general population of community-dwelling adults. The current study sought to address this gap in the literature.

## Methods

### Participants and data collection

The current validation study is part of a larger study that aimed to explore quality of life in Greece following the economic crisis [9]. The Institutional Review Board (IRB) approval was obtained from Springfield College. Next, two independent, professional translators - whose mother tongue was Greek-translated the MQLI from English to Greek according to cross-cultural adaptation procedures for self-report measures [21]. Translations were compared for discrepancies and translated back to the original language to ensure content validity. Discrepancies included minor wording clarifications; however, no changes were required in the conceptual content. The instrument was checked for semantic equivalency and a *pilot test* was conducted (*N* = 15). Based on *pilot test* feedback, revisions were made to the *MQLI-Gr*. It was reported to be easy to use and took about 3 min to complete. The survey was administered in-person and online (Survey Monkey) to Greek adults in a major Greek city. In order to recruit for online participants, a link to the study was posted on social media pages. Participants had to be 18-years or older, of Greek origin, and have lived in Greece for at least 5 yr [9]. To prevent participants from taking the survey twice they were able to access the link only once. That is, as participants completed the survey the system itself would not permit a second try. Recruitment for in-person participants took place in a central area of a large city in Greece. The research team using convenience sampling approached persons and requested their participation in the QoL study. If the participant agreed, the researcher administered the survey. The requirement to take the survey only once was reiterated at the time in-person surveys were administered.

After reviewing the purpose of the study, the risks/benefits of participation, and given assurances of confidentiality, participants provided informed consent. The following instruments were administered: (a) *Multicultural Quality of Life Index*-*Greek version* (*MQLI-Gr*), (b) *Depression Anxiety Stress Scales* (DASS-42), and (c) *Brief Survey Form* which included the demographic variables marital status, income, and employment [9].

### Measurements

*Depression Anxiety Stress Scale* (DASS-42) is a 42-item, self-report scale which provides separate scores for depression, anxiety, and stress. Each scale consists of 14 items rated on a 3-point Likert scale. The maximum score for each of the three subscales is 42 with higher scores indicating more severe symptoms. The higher the score the more severe the affective state [22]. The following scoring was based on reports by the DASS-42 authors and was used to differentiate two groups of respondents. One group reported *none to mild symptoms* of Depression (0–9), Anxiety (0–7) and Stress (score 0–14), the other group reported *severe symptoms* of Depression (28+) Anxiety (20+), and Stress (34+). DASS-42 takes about 10 min to complete and demonstrates strong test-retest reliability and construct validity [22–25]. DASS-42 has been validated in several languages including Greek [25–27] (Table 1).

**Table 1:** Multicultural quality of life index (MQLI-Gr)

Multicultural quality of life index-greek translation Πολυπολιτισμικός δείκτης ποιότητας ζωής Οδηγίες: Προσδιορίστε εδώ την ποιότητα της υγείας και ζωής σας, από Καθόλου έως Εξαιρετικά, βάζοντας X σε ένα από τα δέκα σημεία για καθεμιά από τις παρακάτω ερωτήσεις:										
1) **Σωματική ευεξία** (αισθάνεστε ενεργητικοί χωρίς πόνους και άλλα σωματικά προβλήματα)										
Καθόλου					Εξαιρετικά					
1	2	3	4	5	6	7	8	9	10	
2) **Ψυχική/ συναισθηματική ευεξία** (αισθάνεστε καλά και άνετα με τον εαυτό σας)										
Καθόλου					Εξαιρετικά					
1	2	3	4	5	6	7	8	9	10	
3) **Προσωπική φροντίδα και ανεξαρτησία** (εκπληρώνετε με ευκολία τις καθημερινές σας ανάγκες λαμβάνετε με ευκολία τις αποφάσεις που απαιτούνται σε κάθε περίπτωση)										
Καθόλου					Εξαιρετικά					
1	2	3	4	5	6	7	8	9	10	
4**) Επαγγελματική δραστηριότητα** (εκπληρώνετε με ευκολία τα καθήκοντά σας στην εργασία το σχολείο το σπίτι)										
Καθόλου					Εξαιρετικα					
1	2	3	4	5	6	7	8	9	10	
5**) Διαπροσωπικές σχέσεις** (είστε ικανοί να ανταποκριθείτε και να διατηρείτε καλές σχέσεις με την οικογένεια τους φίλους και ομάδες ατόμων)										
Καθόλου					Εξαιρετικα					
1		2	3	4	5	6	7	8	9	10
6) **Κοινωνική και συναισθηματική υποστήριξη** (υπάρχουν στη ζωή σας άνθρωποι τους οποίους εμπιστεύεστε και οι οποίοι μπορούν να σας προσφέρουν βοήθεια και συναισθηματική στήριξη)										
Καθόλου					Εξαιρετικά					
1	2	3	4	5	6	7	8	9	10	
7) **Υποστήριξη από τον δήμο/κοινότητα και υπηρεσίες** (ευχάριστη και ασφαλής γειτονιά πρόσβαση σε οικονομικές πληροφοριακές και άλλες πηγές/ υπηρεσίες)										
Καθόλου					Εξαιρετικα					
1	2	3	4	5	6	7	8	9	10	
8) **Προσωπική ολοκλήρωση** (βιώνετε αίσθηση ισορροπίας		αξιοπρέπειας και αλληλεγγύης			απολαμβάνετε την σεξουαλικότητά σας			τις τέχνες κ.λπ.)		
Καθόλου					Εξαιρετικα					
1	2	3	4	5	6	7	8	9	10	
9) **Πνευματική ολοκλήρωση** (βιώνετε πίστη θρησκευτικότητα και υπέρβαση της συνηθισμένης υλικής ζωής)										
Καθόλου					Εξαιρετικα					
1	2	3	4	5	6	7	8	9	10	
10) **Αντίληψη της ποιότητας ζωής** (αισθάνεστε ικανοποιημένοι και ευτυχισμένοι με τη ζωή σας γενικά)										
Καθόλου					Εξαιρετικά					
1	2	3	4	5	6	7	8	9	10	

### Analysis

The Statistical Package for the Social Sciences (SPSS) version 24 and the M*plus* program were used for data analysis. The default estimator for CFA in M*plus* is WLSMV (diagonally weighted least squares) and is appropriate for ordinal level data. Next, data were cleaned and checked for anomalies and frequencies run to check the accuracy of each variable. To assess internal reliability, internal structure, and construct validity, the following analyses were conducted: Cronbach’s α coefficient, confirmatory factor analysis (CFA), independent t-test, and ANOVA. A confirmatory factor analysis (CFA) [28] was conducted to evaluate a one-factor model (personal satisfaction) [18]. The following four indexes along with cut-off criteria [29] were used to assess model fit: the model Chi-Square [cut-off *p* > .05]; the comparative fit index (CFI) [cut-off > .90]; the root mean square error of approximation (RMSEA) [cut-off < .05 represents close approximate fit, results between .05 and .08 suggest reasonable error of approximation, and > .10 indicates poor fit] [29]; and the standardized root mean square residual (SRMR) [cut-off < .08].

## Compliance with ethical standards

The authors declare there are no conflicts of interest. All procedures involving human participants were in accordance with the ethical standards of the institutional and/or national research committee and with the 1964 Helsinki declaration and its later amendments or comparable ethical standards. Informed consent was obtained from all participants in the study.

## Results

A convenience sample of one thousand and sixty-four community-dwelling Greek adults (*N* = 1064) participated in the survey. Only surveys where participants completed the *MQLI-Gr* were used resulting in a net sample size of 884 participants. Fifty-two percent of the participants (*n* = 455) completed the survey in-person, and 429 participants completed the survey online.

Ages ranged from 18 to 92-years-old (M = 37.1, SD = 12.9), all participants were Greek and the majority were female (66.2%) with a mean income €625. It should be noted that women were over-represented at 66.2%, whereas the Greek population is 50% female. The average score for the MQLI-Gr was a mean of 6.5 with a standard deviation (SD) of 1.7. Descriptive statistics of individual items on the *MQLI-Gr* are presented in Table 2.

**Table 2:** Descriptive statistics of the MQLI-Gr

Items	Mean	SD	Skewness	Kurtosis	Alpha if item deleted	Corrected item-Total correlation
MQLI 1	6.78	2.21	−.496	−.390	.891	.701
MQLI 2	6.54	2.42	−.492	−.579	.884	.796
MQLI 3	6.77	2.36	−.603	−.419	.888	.737
MQLI 4	6.97	2.39	−.788	−.166	.891	.687
MQLI 5	7.59	2.04	−.988	.590	.888	.764
MQLI 6	7.97	2.17	−1.312	1.293	.900	.544
MQLI 7	4.05	2.68	.375	−1.096	.916	.327
MQLI 8	6.50	2.47	−.579	−.579	.885	.780
MQLI 9	6.00	2.63	- .338	−.936	.901	.543
MQLI10	6.11	2.37	−.477	−.644	.885	.782

### Internal consistency and internal structure

The *MQLI-Gr* showed high internal consistency (Cronbach’s alpha = .90). Table 3 presents the results of four fit indexes achieved by the one-factor model tested by means of confirmatory factor analysis (CFA). Results of two indexes did not meet the criteria for good fit: the *X*^*2*^ statistic (criteria *p* > .05, result = *p* < .001), and the RMSEA statistic (criteria <.08, result = .16). Two fit indexes met the criteria for good model fit: the CFI statistic (criteria >.90, result = .95), and the SRMR statistic (criteria <.08, result = .03). Correlated errors were also run; however, there were no substantive improvements in the model fit indices. Factor loadings on each of the individual items of *MGLI-Gr* were .56 and above except *Community and Support Services* (.31) [see Table 4.].

**Table 3:** Confirmatory factor analyses for ordinal data (*N* = 884)

Fit Index	1 Factor
Model Chi-Square (df)	846.75 (35)^a^
Comparative fit index (CFI)	.950
Root mean square error of approximation (RMSEA)	.116
Standardized root mean square residual (SRMR)	.032

^*a*^*df* degrees of freedom

*p < .001*

**Table 4:** Factor loadings for MQLI-Gr (*N* = 884)

Items	Factor loadings
Physical Well–being	.77
Psychological./Emotional Well-being	.87
Self-Care/Indep. Functioning	.81
Occupational Functioning	.75
Interpersonal Functioning	.80
Social-Emotional Support	.59
Community and Services Sup.	.31
Personal Fulfilment	.84
Spiritual Fulfilment	.60
Global Perception of QoL	.81

### Convergent validity

Pearson correlation tests were run on depression, anxiety, stress (DASS-42) and *MQLI-Gr*. Results showed an inverse relationship with depression (*r* = −.63, *p* < 0.001), 95% CI [.59, .67] anxiety (*r* = −.50, *p* < 0.001), 95% CI [.44, .55] and stress (*r* = −.51, *p* < 0.001), 95% CI [.45, .55] and the higher the score on depression, anxiety and stress, the lower the *MQLI-Gr* score.

### Discriminant validity

DASS-42 was used to assess discriminant validity. Independent t-tests were run to compare the means of two groups hypothesized to differ in quality of life-those with *none-mild symptoms* versus those with *severe* symptoms of depression, anxiety, and stress. For all three subscales, there was a statistically significant difference in *MQLI-Gr* scores between the two groups, with the *mild group* scoring higher on *MQLI-Gr* [indicative of better quality of life] as compared to the *severe group.* Results are as follows: **D***M* = 3.04, 95% CI [2.6, 3.4], *t* (102.43) = 14.99, *p* = 0.001; **AN***M* = 2.34, 95% CI [1.9, 2.7], *t* (133.56) = 12.27, *p* = 0.001; **S***M* = 2.45, 95% CI [1.9, 3.0], *t* (63.98) = 8.882, *p* = 0.001.

To further assess the ability of *MQLI-Gr* to discriminate between groups hypothesized to differ on *quality of life,* marital status, income, and employment were examined [3, 9, 30–32]. Because the Levine test of homogeneity was violated for each variable (*p* < .05), the Welch ANOVA with post hoc Games-Howell test was used to interpret the results. The following results were statistically significant at *p* < .05 level. Results indicated those who reported *single* as compared to *married* were more likely to report lower *quality of life*; those who *earned less than €499* were more likely to report lower *quality of life*; and *students-unemployed* were more likely to report lower *quality of life* [see Table 5].

**Table 5:** Discriminant validity: MQLI-Gr marital, income, & employment status

		Mean	Levine	ANOVA WELCH ***(p*** ***<*** ***0.05)***	^a^Post hoc ***(p*** ***<*** ***0.05)***
Marital status (*N* = 859)					
Single	271	**(6.3)**^a^			
Relation/engaged	241	(6.4)	*p* = 0.05	*F* (2, 534.61) = 6.15,	GH (.46, 95% CI (.14, .80)
Married	347	(6.8)			
Income Level (*N* = 596)					
<€499	268	**(6.4)**^a^			
€500–999	196	(6.7)	*p* = 0.02	*F* (2,347.191) = 3.962,	GH (.48, 95% CI (.69, .89)
€ 1000>	132	(6.9)			
Employment status (*N* = 765)					
Employed	484	(6.8)			
Stud./unempl	*85*	(6.6)	*p* = 0.05	*F* (2,214.003) = 9.6,	GH (.66, 95% CI (.31, 1.0)
Unemployed	196	**(6.1)**^a^			

^a^*Note:* Participants who were *single* were statistically significantly more likely to report lower QoL; those who earned *less than €499* were more likely to report lower QoL; the *students-unemployed* group were more likely to report lower QoL (*p* < .05)

## Discussion

The current validation study demonstrates the psychometric properties of the *MQLI-Gr*. The MQLI has been validated in several languages and researchers [10, 17–20] have noted that a significant strength of the MQLI is cultural relevance. We believe the current study contributes to cultural relevance by the fact that the authors have intentionally paid close attention to the cultural and linguistic precision of each item. In terms of time efficiency, and consistent with the literature [10, 16–20], participants in the *pilot test* reported *MQLI-Gr* was easy to understand and took only a few minutes to complete.

Congruent with earlier validation studies of MQLI [16–20], this study demonstrated good internal consistency. An examination of the internal structure of the MQLI-Gr was not definitive; however, with model fit indices achieving mixed results. We note that the large sample size (*N* = 884) along with non-normality (ordinal level data) of the current study may have contributed to the difficulty of achieving good model fit with the Model Chi-Square. Factor loadings on *MQLI-Gr* items were strong with the exception of *Community and Services Support* (.31) which was comparatively low. This latter result may be understood in the context of the financial crisis which compromised the availability of community services and supports in Greek communities [32]. This result is also consistent with the Organization for Economic Co-operation and Development (OECD) Better Life Index reports wherein Greek citizens indicated dissatisfaction with the public sector and community services [9, 33]. With respect to discriminant validity our results were similar to prior research [18]. Specifically, *quality of life* was statistically significantly different and lower (*p* < .05), for participants with severe depression, anxiety and stress as compared those who with mild symptoms. Also consistent with the literature, discriminate validity was demonstrated on groups thought to differ on quality of life [34–37]. Specifically, persons who were unemployed, single, and without a steady source of income reported poor *quality of life* as compared to persons who were employed, married and with a steady source of income.

### Limitations

This was the first step in the validation of a *quality of life* measure for community-dwelling Greek adults. As in any beginning endeavor, there were a number of limitations. As this was a non-representative sample, results should be interpreted cautiously. The use of non-probability, as opposed to probability sampling, was a significant limitation. Persons who self-selected to participate in the *quality of life* survey may not represent most Greek adults, and thus results are prone to biased estimates. Convenience sampling also prohibits generalizing beyond the sample, and thus results may be limited in overall applicability. Secondly, the use of online and in-person data collection may have caused ‘mode effects’ for which we did not control. As well, some variables such as *Income* had a high level of ‘missing values’ and we were not able to account for this non-response. Had we incorporated additional ‘missing value’ categories this may have been avoided. Another limitation was in terms of the internal structure of the MQLI-Gr where only two out of four indices indicated good model fit. Therefore, we recommend further work be done to strengthen internal structure--perhaps by continuing to explore the idea of a two-factor model. Finally, with additional tests our validation study of *MQLI-Gr* would have been strengthened. For example, a *test-retest* reliability procedure would have provided an indication of stability and reproducibility, and similarly, an assessment of *criterion validity* would have strengthened the validation of *MQLI-Gr.*

## Future validation studies

In order to strengthen confidence in the efficacy of the *MQLI-Gr*, future investigators should examine the effectiveness of *MQLI-Gr* among a representative sample of Greek adults by using a probability sampling procedure. Future research must consider *test-retest* reliability to assure measurement stability over time and reproducibility and, in addition, consider strengthening the *MQLI-Gr* by incorporating an assessment of *criterion validity*. Future investigators should also explore why the component loadings of *Community and Services Support* item of the *MQLI-Gr* differed as compared to other translated QoL measures. Finally, as there is some concern DASS-42 may tap one, rather than three dimensions, a further exploration of discriminate validity is called for using measures in addition to DASS-42.

## Conclusions

The current study is the first Greek translation and validation of the *MQLI* in a general population of community-dwelling adults. This validation study is a step towards the development of a psychometrically sound measure to assess *quality of life* in Greece. Furthermore, as noted earlier, by paying close attention to the cultural and linguistic precision of each item in the *MQLI-Gr*, the current study contributes to the validation of a culturally relevant *quality of life* measure.

## Data Availability

The database supporting the conclusions of this article is included within this article and its additional files.

## Abbreviations

MQLI: Multicultural Quality of Life
DASS-42: Depression Anxiety Stress Scales
CFA: Confirmatory Factor Analysis
IRB: Institutional Review Board
